# The intention to use an electronic health record and its antecedents among three different categories of clinical staff

**DOI:** 10.1186/s12913-018-3022-0

**Published:** 2018-03-21

**Authors:** Claudio Vitari, Roxana Ologeanu-Taddei

**Affiliations:** 1 IAE Paris 1 Panthéon-Sorbonne (Sorbonne Business School), 8 bis rue de la Croix Jarry, 75013 Paris, France; 2 Montpellier Research in Management, University of Montpellier, 34090 Montpellier, France

**Keywords:** Intention to use, Electronic health record, Clinical staff, Survey, Physicians, Paraprofessionals, Administrative personnel

## Abstract

**Background:**

Like other sectors, the healthcare sector has to deal with the issue of users’ acceptance of IT. In healthcare, different factors affecting healthcare professionals’ acceptance of software applications have been investigated. Unfortunately, inconsistent results have been found, maybe because the different studies focused on different IT and occupational groups. Consequently, more studies are needed to investigate these implications for recent technology, such as Electronic Health Records (EHR).

**Methods:**

Given these findings in the existing literature, we pose the following research question: “To what extent do the different categories of clinical staff (physicians, paraprofessionals and administrative personnel) influence the intention to use an EHR and its antecedents?” To answer this research question we develop a research model that we empirically tested via a survey, including the following variables: intention to use, ease of use, usefulness, anxiety, self-efficacy, trust, misfit and data security. Our purpose is to clarify the possible differences existing between different staff categories.

**Results:**

For the entire personnel, all the hypotheses are confirmed: anxiety, self-efficacy, trust influence ease of use; ease of use, misfit, self-efficacy, data security impact usefulness; usefulness and ease of use contribute to intention to use the EHR. They are also all confirmed for physicians, residents, carers and nurses but not for secretaries and assistants. Secretaries’ and assistants’ perception of the ease of use of EHR does not influence their intention to use it and they could not be influenced by self-efficacy in the development of their perception of the ease of use of EHR.

**Conclusions:**

These results may be explained by the fact that secretaries, unlike physicians and nurses, have to follow rules and procedures for their work, including working with EHR. They have less professional autonomy than healthcare professionals and no medical responsibility. This result is also in line with previous literature highlighting that administrators are more motivated by the use of IT in healthcare.

## Background

While the implementation, diversity and uses of information technology (IT) have increased in hospitals, previous research has highlighted its low use among physicians and their resistance to it [1, 2]. Thus the healthcare sector has to deal with the issue of users’ acceptance of IT. Acceptance is now considered a mature field in information systems research [3]. The model assumes that two main antecedents—perceived ease of use (PEOU) and perceived usefulness (PU)—influence the intended and actual use of new IT. PEOU is “the degree to which a person believes that using a particular system would be free from effort” [4]; PU is “the degree to which a person believes that using a particular system would enhance his or her job performance” [4].

In healthcare, different factors affecting healthcare professionals’ acceptance of software applications have been investigated. Unfortunately, inconsistent results have been found [5–7], maybe because the different studies focused on different IT and different occupational groups. Consequently, more studies are needed to investigate these implications for recent technology, such as electronic health records (EHR).

While previous research showed the effect of task characteristics as moderate variable [8], very little research has been done on IT acceptance for different occupational groups in the health context. Generally, studies select one occupational group or healthcare staff as a whole [9–11]. These studies fail to acknowledge the diversity in the autonomy, work practices and power of the occupational groups involved in patient care. There are three main professionals groups within hospitals: doctors, paraprofessionals and administrative personnel [12]. The people in each of these three groups have different educational backgrounds and professional cultures that play a key role in shaping their attitudes toward technologies in the workplace. While doctors tend to be more unwilling to change their traditional practice and use new IT, paraprofessionals tend to accept and use them more readily [13]. Moreover, paraprofessionals adopt more favorable attitudes toward new technology than doctors because they are likely to perceive it as an effective tool for facilitating coordination with other healthcare groups, another important role for them in addition to their bedside responsibilities [12, 14]. Some authors consider that doctors, unlike paraprofessionals, may perceive new IT as a threat because they lead to loss of power [12], challenging professions and driving to a “new professionalism” [15]. Administrative personnel seem to take yet another point of view. In general, they coordinate external and internal activities, for example, doctors’ and paraprofessionals’ schedules, to optimize healthcare operations [12]. They generally have a favorable attitude toward the adoption and use of new IT in healthcare because it helps them monitor and manage the work of doctors and paraprofessionals [12, 16].

Given these findings in the existing literature, we aim to measure PEOU and PU for different occupational groups (doctors, paraprofessionals and administrative personnel) in the same hospital setting, relative to adoption of EHR.

We pose the following research question: “To what extent do the different categories of clinical staff (physicians, paraprofessionals and administrative personnel) influence the intention to use an EHR and its antecedents?”

**Fig. 1:**
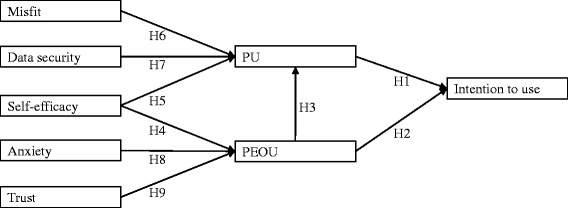
The theoretical model. Detailed legend: Each rectangle is a construct, each arrow is a hypothesis

To answer this research question we develop a research model (Fig. 1), with the following hypotheses that we empirically tested via a survey.

The measure of intention of use and its antecedents were initially developed for voluntary uses of IT. Nevertheless, based on previous literature [17, 18] intention to use was also measured in mandatory settings [19, 20], according to the assumption that “even when users perceive system use to be organizationally mandated, usage intentions vary because some users are unwilling to comply with such mandates” [19, 21]. Consequently Venkatesh & Davis [19] defined voluntariness as “the extent to which potential adopters perceive the adoption decision to be non-mandatory”. This definition highlights the difficulty of the degree of voluntarism involved [22, 23] because “even when use is required, variability in the quality and intensity of this use is likely to have a significant impact on the realization of the system benefits” [22]. Even in mandatory settings, the extent of system use varies by user [24–26] and can lead to users’ resistance, which is more or less passive [27], and to workarounds [28]. Thus, the concept of intention of use is relevant because it can assess the willing to really use the system or to find alternative ways as workarounds.

Studies on intention to use and its antecedents in healthcare highlight relationships that differ from those in other sectors. For example, a study on a clinical decision support system showed that the influence of PU on technology acceptance among physicians was significantly supported, but the influence of PEOU was not [5]. In addition, a systematic review of the literature on physician acceptance of IT [6] showed that the PEOU component of the model is consistently related to PU. This finding is consistent with the results of Holden and Karsh [29] who reviewed 16 data sets. Every one of the 16 tests of the relationship between PU and intention to use was significant. While Aggelidis and Chatzoglou [30] and Pai and Huang [31] state that PEOU affects intention to use, Chau and Hu [5] show that PEOU has no significant effect on PU or physicians’ perceptions. Some authors suggest that this inconsistency in the results may be explained by the rapid grasp of the importance of usefulness as opposed to usability [29, 32–34], and the availability of support staff to deal with the system [35].

The PEOU-intention relationship seems to be more inconsistent. It is significant in only seven of 13 tests of the sets relating to mixed groups of professionals and paraprofessionals Holden and Karsh analyzed [29]. Nevertheless, we consider that the effect of PEOU on intention is more likely to be positive, given the majority of tests and previous studies in different organizational settings. We therefore hypothesize:

- HI. PU is positively related to intention to use.
- H2. PEOU is positively related to intention to use.
- H3. PEOU is positively related to PU.

Research identified different antecedents of PU and PEOU. Several concepts were tested. Their variety is one reason for the difficulty in comparing the results of different studies. Subjective norms seem to be less significant for physicians because of their professional independence. Given the specificity of physicians and all health professionals, we might consider self-efficacy a pertinent antecedent. Self-efficacy is defined as “one’s belief in his or her ability to execute a particular task/job using a computer” [36, 37]. In line with Bandura’s self-efficacy theory [38], authors have shown that individuals with higher self-efficacy are more likely to experience a positive effect than those with lower self-efficacy. Authors identified that computer self-efficacy influences both PEOU and PU [39], while other results [7] find that self-efficacy has no significant effect on either PU or PEOU. We therefore hypothesize:

- H4. Self-efficacy is positively related to PEOU.
- H5. Self-efficacy is positively related to PU.

Prior studies have found that compatibility is an important factor that impacts upon willingness to adopt innovative technology [40]. Generally, the misfit between health IT (including EHR) and work practices [41, 42] is highlighted. At the same time fit is a very challenging objective because of the complexity and variety of clinical processes [43].

Several authors [29, 44, 45] maintain that creating a fit between health IT and existing work practices requires the initial acknowledgment that the former will change the latter. By customizing and adapting the system to meet specific needs, users will become more open to using it [42, 46]. The fit between clinical work tasks and the design of the technology significantly impacts on the likelihood of acceptance of health IT [47]. We therefore hypothesize:

- H6. Misfit is negatively related to PU.

Previous research shows contrasting results about the perceived importance of data security in the provision of healthcare on the PU of health IT [48, 49]. Previous studies have insisted that both privacy and the confidentiality of patient data [42, 50] are necessary to ensure the use of health IT [42]. We consider that data security implies both these factors. We therefore hypothesize:

- H7. Data security is positively related to PU.

Computer anxiety, defined as people’s apprehension, or even fear, when they are faced with the possibility of using computers [37, 51], has also been found to have a significant impact on PEOU in the context of physicians’ acceptance of health IT [49, 52]. We therefore hypothesize:

- H8. Anxiety is positively related to PEOU.

Many studies highlight the importance of trust as a driver of human behavior [53–55]. Trust in healthcare IT may include having confidence in both the providers and the system [56, 57]. Within the healthcare sector, the strong influence of physicians’ trust on PEOU is consistent with earlier studies that show the positive effect of situational normality and structural assurances on PEOU [58]. We therefore hypothesize:

- H9. Trust is positively related to PEOU.

The purpose of this study is to clarify the possible differences, in intention to use an EHR and its antecedents, existing between different staff categories. As described and argued above, this study proposes 9 hypotheses developed based on previous theories and similar empirical studies.

## Methods

To test our model of Fig. 1 we organized a survey based on an online questionnaire that we administered in a French teaching hospital that covers all the clinical specialties and has more than 2500 beds as well as an emergency department. In 2012 the hospital implemented an EHR system according to a “big bang” strategy, aiming to support all departments and specialties within 9 months. This EHR incorporates computerized physicians’ order entries, medical and nursing observations, laboratory test results, medical prescriptions, operating room process management, and planning and billing management. It is a fully integrated system with a range of different modules, including admission, discharge and transfer, computerized provider order entries, treatment planning, resources and appointment scheduling systems, and a clinical data warehouse (CDW). Results from ancillary subsystems (e.g., laboratories, imaging, and pharmacy) are automatically integrated into the EHR as pdf files.

Our survey measured the clinical staff’s perceptions of EHR, using questions derived from a review of previous studies, adapted from different scales: PU [4, 5], PEOU [4, 5], misfit [7], data security [7], anxiety [37], self-efficacy [7, 19], and trust [40]. Each variable was measured using a question and each question was answered using a seven-point Likert scale, with 1 indicating “strongly disagree” and 7 indicating “strongly agree.” For a better understanding of the EHR context and local setting, we conducted four interviews with the two physicians involved in the EHR evolution and customization. Additional data were collected from internal reports.

The presence of complementary data collection, beyond the needs for this study, limited the possibilities to include whole scales to measure our constructs of interest. We limited our data collection to one item for each variable, keeping our construct narrowly defined in order to make the single-item measures sufficient [59–61]. This decision was made as a response to the request of the president of the Delegation of Hospital Information (mandated to configure and customize the EHR for the clinical staff), who commanded the questionnaire and who manage its administration. His argument was that the questionnaire needs to be very short; unless, healthcare professionals would not find time to respond to it. Nevertheless, initially, the questionnaire comprised more items for every construct. The questionnaire was sent for pre-test to a panel of 20 volunteers. Eleven respondents actively contributed in the pre-test, asking to reduce further the survey because the respondents considered that more items to measure the same construct were redundant. Furthermore, considering the responses, we contextualized the meaning of EHR. As suggested by the pre-test respondents, we decided to use the appellation of the software instead of the generic term “EHR”. The questionnaire was developed and administered online by the Delegation of Hospital Information to the care staff, during 1 month. One recall was made. A link inviting the clinical staff to respond to this questionnaire was communicated to them by email and invitations were posted on different staff resting rooms’ and corridors’ walls.

We noted that the intensity of use of the EHR is not a significant issue because each patient’s medical examinations, medical history, prescriptions, planning schedule and administrative documents had to be entered in the EHR. Thus, all clinical staff had to post information or use information posted by others in their daily work, eventually in different ways.

To take these differences into account, we grouped respondents into three main categories: (1) professionals (all physicians and medical residents in all medical domains); (2) paraprofessionals (all carers and nurses); and (3) administrative personnel (all secretarial staff and personal assistants).

The survey was administrated twice, the first time in December 2013 and then in September 2015. The 21-month lag between the two surveys was decided empirically with the hospital’s top management during data collection, following an important change in the version of EHR used and to avoid summer holidays. As well as our survey questions, the questionnaire included sections relating to several hospital management objectives. All the data were provided by mandatory answers to a questionnaire by the employees of the hospital. We followed strict ethical procedures at every stage of the study to ensure data confidentiality and anonymity of the study participants. We duly explained the purpose of the study to all employees before verbally obtaining their consent to freely participate. All the participants consented to answer anonymously to the questions asked by questionnaire. The survey was presented and accepted by the Medical Commission of the Hospital, the Users’ Commission and the Executive Board of the Hospital. All the data were collected anonymously. The respondents accepted to answer to the questions asked. No other ethics approval was necessary to be compliant with the French regulation.

Between 2013 and 2015 several actions were initiated within the hospital, including training programs for all clinical staff and customization of EHR forms for different medical departments and occupations: the deployment of a new version of the EHR, the development of an e-learning platform, the continuous customization of EHR, the indexing of folders and files in EHR.

We tested our hypotheses using multiple regressions analyses [62] that included the assessment of: (1) the association between intention to use, ease of use and usefulness; (2) the association between usefulness and ease of use, misfit, data security and self-efficacy; (3) the association between ease of use and anxiety, self-efficacy and trust; and (4) the association between intention to use, ease of use, usefulness and control variables (i.e. transverse work, year of questionnaire administration). The regression models were run separately for the three categories (physicians and residents, carers and nurses, secretaries and assistants) and for all three categories together. Where appropriate, differences were also tested using analysis of variance (ANOVA).

## Results

From a population of 6443 clinical employees, we collected 1741 responses with the first distribution of the questionnaire (27% response rate) and 1119 with the second distribution (17% response rate). Concerning our three categories of interest: 169 secretaries and assistants responded to the first questionnaire and 151 the second one, on the 547 total secretaries and assistants employed; 759 carers and nurses participated in the first data collection and 504 in the second one, on the 3754 carers and nurses working at the hospital; 574 physicians and residents contributed to the first questionnaire and 286 to the second one on a total population 1427. The overall percentage of missing data in the sample were small (< 7.5%) and no data imputation was carried out (Tables 1 and 2).

**Table 1:** Variables of the survey

Variable	Question	Mean	SD	Min	Max	N	%^a^
Intention to not use	I prefer to not use the EMR to care for my patients, as far as I can	3.39	2.217	1	7	3139	92.54%
Usefulness	The EMR is useful to care for my patients	4.21	1.728	1	7	3152	92.92%
Ease of use	I find easily the data I need in the EMR	3.55	1.74	1	7	3208	94.58%
Misfit	The EMR misfits with my tasks	4.2	2.108	1	7	3167	93.37%
Data security	The medical records of my patients are secured in the EMR	4.04	1.878	1	7	3142	92.63%
Anxiety	I feel alone facing the EMR	3.75	1.953	1	7	3166	93.34%
Self-efficacy	I have the resources and information to use the EMR to care for my patients	4.05	1.8	1	7	3165	93.31%
Trust	I have trust in the EMR	3.64	1.867	1	7	3167	93.37%

^a^ = The denominator of the % is 3392, the total number of collected questionnaires. The nominator is the N value in the previous column, the total number of responses to the specific question of the survey

**Table 2:** Sample characteristics

		Respondents, for each category, to the questionnaire administered in:			
		Year 2013		Year 2015	
Staff categories	Declaring doing a transversal work	Number of respondents	% of respondents on the total number of respondents	Number of respondents	% of respondents on the total number of respondents
Secretaries and assistants	Yes	16	1.15%	15	1.59%
	No	147	10.53%	136	14.45%
Carers and nurses	Yes	82	5.87%	72	7.65%
	No	630	45.13%	432	45.91%
Physicians and residents	Yes	187	13.40%	113	12.01%
	No	334	23.93%	173	18.38%
Total		1396	100.00%	941	100.00%

Altogether, usefulness and ease of use are good predictors of the intention to use EHR, explaining 34% of the variance, which can be considered moderate [63]. For physicians and residents, 37% of the variance of their intention to use is explained by usefulness and ease of use. We have similar values for carers and nurses (32%). Also, the beta coefficient of the two independent variables for the whole sample and for physicians and residents, as well as carers and nurses, are a similar size, around 0.55 for usefulness and 0.33 for ease of use.

Conversely, medical secretaries and assistants have different intentions and perceptions. For this category of personnel, ease of use and usefulness explain only 19% of the variance of intention to use. While usefulness has a beta coefficient close to that of the other personnel categories (0.46), the ease of use variable has no statistically significant influence on intention to use.

Concerning the antecedents of usefulness and ease of use, we do not identify relevant differences among the different personnel categories. The R-squared of the different independent variables on usefulness is between 0.35 and 0.43, depending on personnel category. The beta coefficients are around 0.29 for ease of use, around 0.13 for self-efficacy, around − 0.18 for misfit (the more EHR fits the job, the more it is perceived as useful), and around 0.17 for data security. The R-squared of the different independent variables on ease of use is between 0.25 and 0.33, depending on personnel category. The beta coefficients are around − 0.10 for anxiety (the less the EHR makes people anxious, the more the people perceive it as ease of use), around 0.15 for self-efficacy, and around 0.36 for trust (Table 3).

**Table 3:** Results of regression analysis split by staff category

Without control variables		Ad.d R2 and *p*-value				β and *p*-value			
Independent variable	Dependent Variable	All sample	Physicians and residents	Carers and nurses	Secretaries and assistants	All sample	Physicians and residents	Carers and nurses	Secretaries and assistants
Usefulness	Intention to not use	0.34***	0.37***	0.32***	0.19***	−0.55***	−0.55***	− 0.54***	−0.46***
Ease of use						−0.31***	− 0.38***	− 0.29***	not sig.
Ease of use	Usefulness	0.39***	0.39***	0.35***	0.42***	0.29***	0.30***	0.27***	0.28***
Self-efficacy						0.11***	0.08**	0.12***	0.17***
Misfit						−0.20***	−0.22***	−0.18***	− 0.14**
Data security						0.18***	0.17***	0.18***	0.16***
Anxiety	Ease of use	0.33***	0.30***	0.33***	0.25***	−0.11***	−0.07*	− 0.12***	− 0.11*
Self-efficacy						0.17***	0.18***	0.17***	0.12*
Trust						0.37***	0.35***	0.37***	0.35***
With control variables		Ad.d R2 and *p*-value				β and *p*-value			
Independent variable	Dependent Variable	All sample	Physicians and residents	Carers and nurses	Secretaries and assistants	All sample	Physicians and residents	Carers and nurses	Secretaries and assistants
Usefulness	Intention to not use	0.35***	0.37***	0.32***	0.20***	−0.54***	−0.54***	−0.54***	−0.44***
Ease of use						−0.31***	−0.38***	− 0.29***	not sig.
Transversal work						not sig.	not sig.	not sig.	not sig.
questionnaire						−0.17*	−0.32*	not sig.	not sig.
Ease of use	Usefulness	0.39***	0.39***	0.35***	0.43***	0.29***	0.30***	0.27***	0.28***
Misfit						−0.20***	−0.22***	− 0.17***	−0.14***
Self-efficacy						0.11***	0.08**	0.12**	0.15***
Data security						0.17***	0.18***	0.18***	0.16***
Transversal work						not sig.	not sig.	not sig.	not sig.
questionnaire						not sig.	not sig.	not sig.	not sig.
Anxiety	Ease of use	0.33***	0.30***	0.33***	0.26***	−0.11***	−0.07*	−0.12***	−0.13*
Self-efficacy						0.17***	0.20***	0.17***	not sig.
Trust						0.38***	0.37***	0.38***	0.37***
Transversal work						not sig.	not sig.	not sig.	not sig.
questionnaire						−0.23***	−0.35**	not sig.	not sig.

*β* Beta coefficient, *Ad.d R2* adjusted R-squared; *** = statistically significant *p*-value lower than 0.001; ** = statistically significant *p*-value lower than 0.01; * = statistically significant *p*-value lower than 0.05; not sig. = not significant when the *p*-value is higher than 0.05

The introduction of control variables in the model helps us to better explain some results and suggest further exploration. We observe substantially stable R-squared across the models and the personnel categories, with few exceptions worth detailing.

Transversal work, i.e. having a job requiring patients’ care in several medical departments, is never statistically significant. The year of questionnaire administration is in some cases statistically significant. For physicians and the personnel as a whole, perception of ease of use of the EHR is negatively influenced by the year of administration of the questionnaire. This suggests that the EHR became more complex to use over time, in parallel with the specific initiatives to improve it. These initiatives to improve it could also explain the influence of the year of the questionnaire administration on the intention to use the EHR for the same physicians and the personnel as a whole: the year of the questionnaire administration positively impacts the intention to use the EHR. The initiatives to improve the EHR made it more difficult to use, but the physicians and the personnel altogether recognized the importance of using it and hence they strengthen their intention to use the EHR.

On the opposite, year of administration did not seem to have any effect on carers, nurses, secretaries and assistants, except making not significant the influence of self-efficacy on ease of use for secretaries and assistants.

Finally, for all the variables, the three personnel categories and all categories together have statistically different mean values (Table 4).

**Table 4:** Results of the analysis of variance

Variable	Staff Category								F (sig.)
	Physicians and residents		Carers and nurses		Secretaries and assistants		All		
	Mean	SD	Mean	SD	Mean	SD	Mean	SD	
Trust	3.34	1.84	3.38	1.86	4.42	1.73	3.50	1.87	44,9***
Self-efficacy	3.73	1.72	4.02	1.80	4.89	1.62	4.03	1.79	49,4***
Anxiety	4.07	1.91	3.80	1.97	3.03	1.81	3.80	1.96	33,0***
Ease of use	3.38	1.68	3.30	1.70	4.30	1.61	3.46	1.72	46,5***
Misfit	4.52	2.06	4.50	2.11	3.05	1.87	4.32	2.12	69,0***
Data security	3.86	1.83	3.89	1.94	4.46	1.79	3.95	1.89	13,1***
Usefulness	4.11	1.68	3.86	1.76	4.93	1.40	4.08	1.72	49,0***
Intention to not use	3.78	2.26	3.66	2.25	2.19	1.60	3.52	2.24	65,4***

*SD* Standard Deviation = F-test of the ANOVA; *** = statistically significant *p*-value lower than 0,001

## Discussion

Our results enrich previous contrasting results on intention to use and its antecedents for health IT. We highlight some differences among personnel categories, an aspect that is still largely unclear in the literature.

For the whole personnel together, all the hypotheses are confirmed, as they are for physicians, residents, carers and nurses. Results are different for secretaries and assistants, whose intention to use the EHR is only influenced by perceived usefulness. Moreover, secretaries and assistants could not be influenced by self-efficacy in the development of their perception of ease of use of the EHR, while for the other personnel categories, self-efficacy, anxiety and trust influence the perceived ease of use of the EHR. We see a considerable difference between physicians, residents, carers and nurses, on the one hand, and secretaries and assistants on the other. For this staff category ease of use does not have any influence on intention to use. This result may be explained by the fact that secretaries, unlike physicians and nurses, are administrative employees and, consequently, have more stringent rules and procedures for their work, including work with the EHR, and they have less professional autonomy. Moreover, secretaries have no medical responsibilities related to patient care and their risk of errors is in the form of errors in recording or failing to find relevant information.

While the literature highlights differences between physicians, residents, carers and nurses in the acceptance and use of a new IT [12–14], we see a certain homogeneity among these categories. The differences are with the third personnel category— secretaries and assistants—as already identified by some researchers [12, 16].

Comparing our results with previous literature [9, 31] we reaffirm, contrary to earlier results [5, 6], that for physicians perceived ease of use has a moderate influence on perceived usefulness. Again, we confirm the role of self-efficacy in building perceptions of usefulness [39], while questioning its role on ease of use perceptions, in the open debate about their effects and the contrasting empirical results [7].

Another open debate concerns the role data security plays in perceived usefulness. While some previous results affirm that data security has an impact [48], and others do not [49], our results demonstrate that data security has a positive influence on the perceived usefulness of EHR.

Notwithstanding the useful results, this analysis has some limitations. The anonymity of the respondents did not allow us to precisely measure the evolution of the perceptions for each single respondent, via a longitudinal study. Moreover, our survey targeted only one hospital for data collection, which limits the generalizability of our findings. Additionally, we restricted the measure of each construct, in the questionnaire, at one single item. The use of full scales, at the place of single items, would have increased the explained variance and opened to additional statistical analysis.

## Conclusions

While the existing literature has focused on the acceptability of health IT by an occupational group or by healthcare staff as a whole, our paper investigates the differences between occupational groups within a teaching hospital. More concretely, we measure the extent to which the three largest categories of clinical staff (physicians, paraprofessionals and administrative personnel) have an influence on the intention to use an EHR and its antecedents.

Mobilizing a set of antecedents of intention to use, we propose a model based on nine hypotheses. The hypotheses were tested mainly using multiple regressions analyses, with and without control variables. For the entire personnel, as a whole, all the hypotheses are confirmed. They are also all confirmed for physicians, residents, carers and nurses but not for secretaries and assistants. Unlike the other personnel categories, secretaries’ and assistants’ perception of the ease of use of EHR does not influence their intention to use it. Moreover, secretaries and assistants could not be influenced by self-efficacy in the development of their perception of the ease of use of EHR. These results may be explained by the fact that secretaries, unlike physicians and nurses, have to follow more stringent rules and procedures for their work, including working with EHR. They have less professional autonomy than healthcare professionals and no medical responsibility.

We suggest that hospital managers have to take these differences among staff categories in a hospital into account when they implement health IT, particularly in light of the interdependence of these staff categories in patient care and the need to involve all categories in the use of the technology.

We hope that our results would contribute to help EHR decision makers and developers to understand the specific motives impacting the use of the EHR for the different staff categories and hence to include these specificities in their development plans. In particular, secretaries and assistants would need special attention, as their antecedents to the intention to use an EHR have a distinct impact in comparison to the impact that the same antecedents have on the other staff categories. In our study, we advance some justifications for these differences, basing on literature and our empirical knowledge. Nonetheless, we consider that future studies should extend our understanding of the origin of these differences, through qualitative and quantitative methods.

## Abbreviations

CDW: Clinical data warehouse
EHR: Electronic Health Records
IT: Information technology
PEOU: Perceived ease of use
PU: Perceived usefulness
